# Digital pathology-based artificial intelligence models for differential diagnosis and prognosis of sporadic odontogenic keratocysts

**DOI:** 10.1038/s41368-024-00287-y

**Published:** 2024-02-26

**Authors:** Xinjia Cai, Heyu Zhang, Yanjin Wang, Jianyun Zhang, Tiejun Li

**Affiliations:** 1 Department of Oral Pathology, Peking University School and Hospital of Stomatology & National Center of Stomatology & National Clinical Research Center for Oral Diseases & National Engineering Research Center of Oral Biomaterials and Digital Medical Devices, Beijing, China; 2 Central Laboratory, Peking University School and Hospital of Stomatology, Beijing, China; 3 Department of Oral Pathology, Shanghai Stomatological Hospital & School of Stomatology, Fudan University, Shanghai, China; 4 Research Unit of Precision Pathologic Diagnosis in Tumors of the Oral and Maxillofacial Regions, Chinese Academy of Medical Sciences (2019RU034), Beijing, China

**Keywords:** Translational research, Oral diseases

## Abstract

Odontogenic keratocyst (OKC) is a common jaw cyst with a high recurrence rate. OKC combined with basal cell carcinoma as well as skeletal and other developmental abnormalities is thought to be associated with Gorlin syndrome. Moreover, OKC needs to be differentiated from orthokeratinized odontogenic cyst and other jaw cysts. Because of the different prognosis, differential diagnosis of several cysts can contribute to clinical management. We collected 519 cases, comprising a total of 2 157 hematoxylin and eosin-stained images, to develop digital pathology-based artificial intelligence (AI) models for the diagnosis and prognosis of OKC. The Inception_v3 neural network was utilized to train and test models developed from patch-level images. Finally, whole slide image-level AI models were developed by integrating deep learning-generated pathology features with several machine learning algorithms. The AI models showed great performance in the diagnosis (AUC = 0.935, 95% CI: 0.898–0.973) and prognosis (AUC = 0.840, 95%CI: 0.751–0.930) of OKC. The advantages of multiple slides model for integrating of histopathological information are demonstrated through a comparison with the single slide model. Furthermore, the study investigates the correlation between AI features generated by deep learning and pathological findings, highlighting the interpretative potential of AI models in the pathology. Here, we have developed the robust diagnostic and prognostic models for OKC. The AI model that is based on digital pathology shows promise potential for applications in odontogenic diseases of the jaw.

## Introduction

Odontogenic keratocyst (OKC) is a cyst that is primarily located in the mandible and mostly affects males in their second and third decades of life.^1–3^ Its occurrence accounts for around 12% of all cysts in the jaw, making it the third most common cystic condition.^4^ The histological features of OKC are a palisading basal cell layer, basophilic nuclei of the basal cells distal to the basement membrane and a corrugated superficial stratum corneum.^3^ Treatment methods for OKC comprise enucleation, marsupialization, curettage, resection, among others.^3^ It has been reported that OKC has a high recurrence rate of ~14%–20%.^1,5,6^ The occurrence of multiple OKCs is frequently linked to Gorlin syndrome (GS, also recognized as nevoid basal cell carcinoma syndrome).^2,7^ GS is an unusual autosomal dominant disorder that manifests in various clinical features such as basal cell carcinoma, OKCs, palmar dyskeratosis, and plantar depression.^7^ It arises from mutations in the *PTCH* gene and abnormalities in sonic hedgehog signal, which result in the proliferation of tumor cells.^7^ Therefore, OKCs have been categorized as sporadic OKC and GS-associated OKC, whereby the latter exhibits a significantly higher rate of recurrence compared to sporadic OKC.^5,6,8^ Orthokeratinized odontogenic cyst (OOC) is a rare type of developmental cyst that makes up around 10% of what was previously classified as OKCs but have now been recognized as a separate entity.^9^ OOCs have unique clinical features that set them apart from OKCs, including the absence of *PTCH1* mutation and a lack of tendency to recur.^10,11^ It has been identified that large lesions of OOC may exhibit comparable radiographic traits to those of OKC.^10^ However, OOC presents distinctive and diagnostic features morphologically. The lining of OOC comprises mostly of thin and uniform stratified squamous epithelium and the basal layer cells were flattened or cuboidal in shape.^10^ Additionally, the palisading basal cells with reverse nuclear polarity and detachment of the lining epithelium from the fibrous capsule, which are the histopathological features of OKC, were infrequently observed in OOC.^5,10^ The rates of recurrence differ significantly between OKC, OOC, and GS. Therefore, it is imperative to recognize the three diseases for their early identification and management.

The higher intrinsic growth potential of OKC compared to other types of odontogenic cysts is a key feature that may also contribute to its higher propensity for postoperative recurrence.^2^ Studies have investigated various clinical, imaging, histological, and molecular pathological aspects of risk factors that may be associated with OKC recurrence, but there is still a lack of clinically applicable models that are sufficient to assess the recurrence potential of OKC.^6,12–16^ Histopathological examination of tissue slides is vital for diagnosing OKC. Histopathological slides typically contain hundreds of thousands of cells and artificial intelligence (AI)-assisted analyses of these samples can provide invaluable scientific and medical information.^17^ Recently, an increasing number of studies have employed AI-based image analysis techniques to evaluate routine histological slides.^18–30^ This offers significant potential for disease research, as AI algorithms can support the decision-making of clinicians and pathologists by analyzing numerous digitized histological slides to derive relevant scientific and clinical insights.^17,31^ In addition, AI systems broaden the scope of information that can be derived from histopathology slides. AI enables the identification of differential diagnoses and prediction of prognoses based on histopathological images of hematoxylin and eosin (H&E) stained disease samples.^32^

In this study, we developed two AI systems for constructing diagnostic and prognostic models of OKC using deep learning algorithms. The models exhibited excellent performance on the testing dataset, providing evidence for the potential contribution of AI in the clinical application.

## Results

**Fig. 1:**
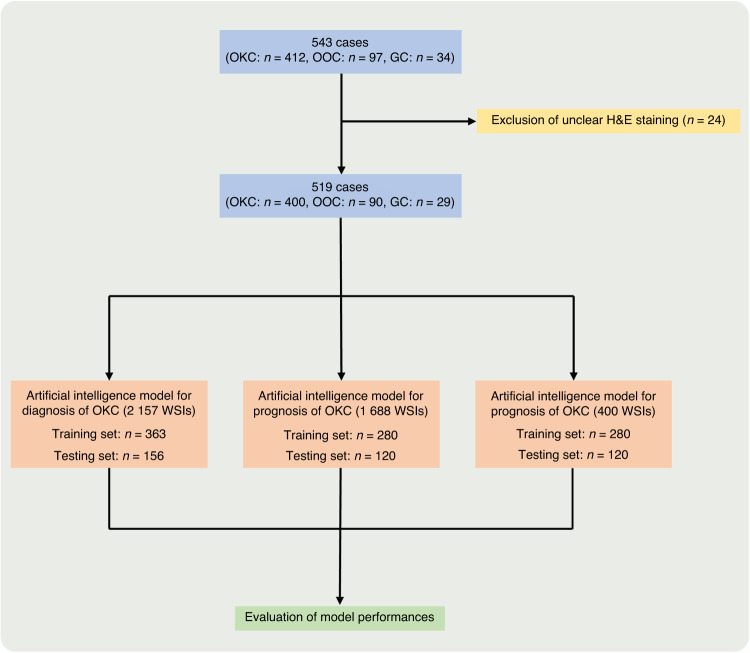
The flowchart for the cohorts used in this study. A total of 543 cases, encompassing OKC, OOC, and GS, were obtained. Of these, 24 cases were excluded due to unclear or faded H&E staining. The remaining 519 cases, along with a total of 2 157 H&E-stained slides, were then randomly assigned to the training and testing cohorts. The training cohort comprised 363 cases, while the testing cohort had 156 cases. Four hundred cases of OKC were randomly assigned into two groups: the training cohort (280 cases) and the testing cohort (120 cases), in order to develop a prognostic model of OKC using a ratio of 7:3

The flowchart for the cohorts used in this study is shown in Fig. 1. The samples were divided into a training cohort, which constituted 70% of the dataset, and a testing cohort, which constituted the remaining 30%, in this study. Fivefold cross-validation was carried out within the training cohort. Additionally, we boosted the algorithm’s efficacy by using the Grid-Search algorithm to identify the optimal set of parameters.

### t-SNE visualization and cross-validation

**Fig. 2:**
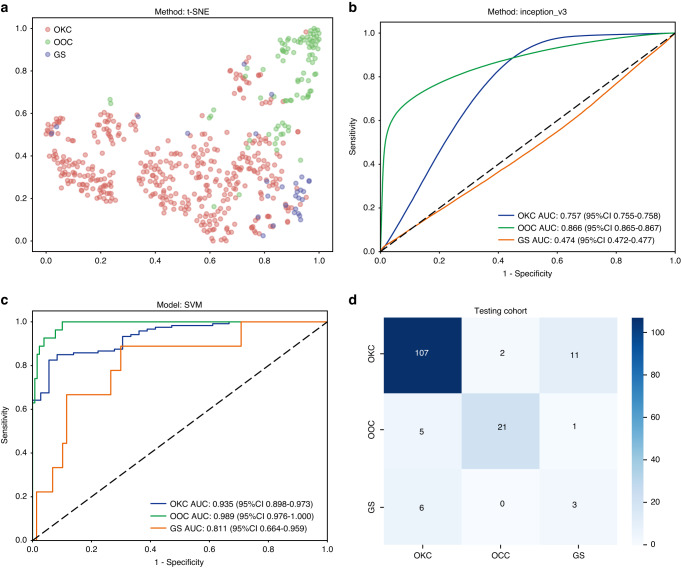
Diagnostic model evaluation. **a** The t-SNE algorithm among three groups of odontogenic keratocyst (OKC), orthokeratinized odontogenic cyst (OOC), and Gorlin syndrome (GS) plotted in a two-dimensional space; **b** The patch-level AUC for diagnosing OKC, OCC, and GS in the testing cohort; **c** The WSI-level AUC for OKC, OOC, and GS in the testing dataset; **d** Confusion matrices for the testing datasets to visually interpret the model classification performance

In the development of the diagnostic model, which included a scenario for three-class classification, the feature dimensionality was limited to a single decimal place. Additionally, we included the predicted labels exclusively as histogram features, resulting in the derivation of a feature set. To understand how patch-level features aggregated into WSI-level representations, we employed the t-SNE algorithm (Fig. 2a). Interestingly, clear differentiation was observed among three groups of OKC, OOC, and GS when plotted in a two-dimensional space. The Grid-Search algorithm was used to determine the optimal model parameters for which parameter tuning was conducted through fivefold cross-validation. Supplementary Fig. 1A depicts the results of our cross-validation on the training dataset, which indicated good performances for all.

### Diagnostic model efficiency

We evaluated the accuracy of the pathology model in region identification by using patch-level ROC curves to compare and characterize the models. Supplementary Table 1 presents the AUC for the model. The patch-level AUC for diagnosing OKC, OCC, and GS in the testing cohort was 0.757 (95% CI: 0.755–0.758, Fig. 2b), 0.866 (95%CI: 0.865–0.867), and 0.474 (95%CI: 0.472–0.477), respectively. To further assess the model performance, we visually evaluated the aggregation of patches into WSI. We acquired predicted labels and probability heatmaps to aid in this assessment. From the results, it is clear that the feature modeling achieved a noteworthy improvement after aggregation through both the BoW and PLH processes. This shows the considerable effectiveness of our feature aggregation logic.

Among all the machine learning methods tested, the SVM algorithm demonstrated the most precise classification outcomes by combining the micro and macro AUCs in the testing cohort (Supplementary Fig. 2). The diagnostic AUC for OKC, OOC, and GS in the testing dataset was 0.935 (95% CI: 0.898–0.973, Fig. 2c), 0.989 (95%CI: 0.976–1.000), 0.811 (95%CI: 0.664–0.959), respectively. Furthermore, we generated confusion matrices for both the training and testing datasets to visually interpret the model classification performance (Supplementary Fig. 1B and Fig. 2d). From the results, it is apparent that there is a tendency for OKC and GS to be confused, while OCC is commonly identified as OCC.

**Fig. 3:**
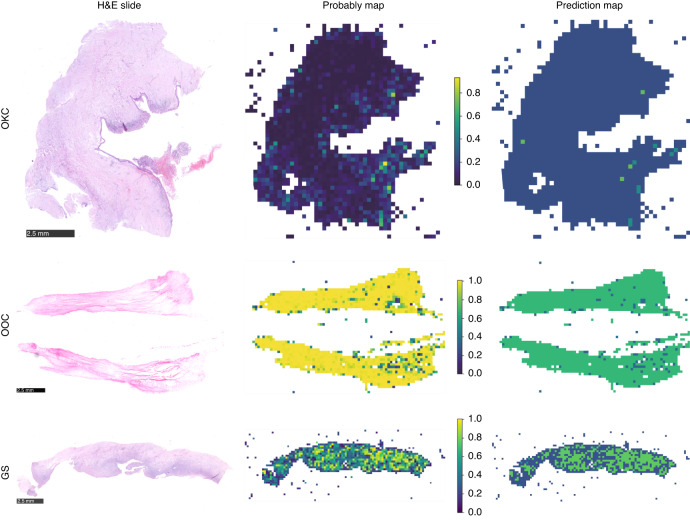
The probably and prediction heatmap of the diagnostic model. The figure shows the H&E slide at the WSI level (left), the heatmap of the predicted probability for each patch (probably map with probability label, middle), and the resulting prediction map of the WSI (right). The OKC is mostly predicted with a probability label of “0”, while the OCC was mostly predicted with a probability label of “1”, and the GS was predicted with a probability label between “0” and “1” and the prediction probability is closer to the OKC

Grad-CAM generates maps that display the localization of classes by visualizing the gradients flowing into the final convolutional layer of the network. This preserves spatial information that is specific to the class. Notably, it does not need any modifications to the model architecture or extra training. Supplementary Fig. 3 illustrates the use of Grad-CAM in displaying the activation of the last convolutional layer for diagnostic class evaluation. This transparent depiction highlights the input image regions that significantly contribute to the prediction, thus providing valuable insights into the decision-making process of the model. As shown in Fig. 3, the probably and prediction heatmap of the diagnostic model clearly indicates that our pathology model achieves a high level of accuracy when assessing region tiles.

### Prognostic model efficiency

**Fig. 4:**
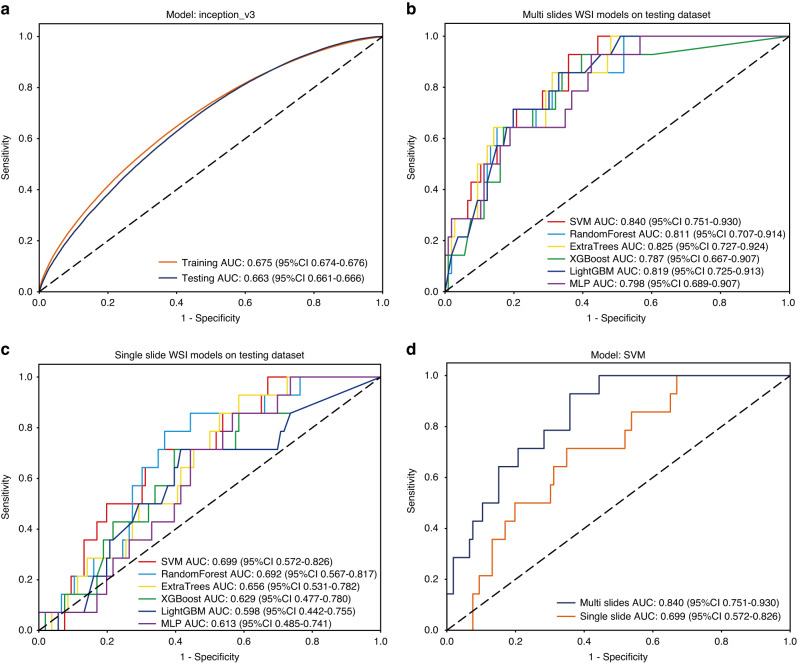
Prognostic model evaluation. **a** the patch-level AUC for the prognostic model in the training and testing cohort; **b** the multiple slides WSI-level AUC for the prognostic model in the testing cohort; **c** the single slide WSI-level AUC for the prognostic model in the testing cohort; **d** the comparison between multiple-slide and single-slide SVM model on the testing dataset

In developing the prognostic model for the recurrence of OKC, we retained two decimal places and integrated both predicted labels and probabilities into the PLH and BoW processes. We performed hyperparameter tuning on the training set using 5-fold cross-validation and the Grid-Search algorithm. The results of this cross-validation are presented in Supplementary Fig. 4A, all of which demonstrate good performance. In the patch-level model for predicting OKC recurrence, the Inception_v3 model displays moderate performance with AUC values of 0.675 (95% CI: 0.674–0.676) for the training dataset and 0.663 (95% CI: 0.661–0.666) for the testing dataset, as illustrated in Fig. 4a.

In addition, we evaluated the aggregation of patches into WSI levels to assess the performance of the models. There were better performances of all machine learning models on the training and test datasets (Table 1), and the SVM model (0.840, 95% CI: 0.751–0.930) and ExtraTrees model (0.825, 95% CI: 0.727–0.924) showed great efficiency on the test dataset (Fig. 4b). This demonstrates that the WSI level leads to better prediction performance compared to the patch level predictions. As shown in Supplementary Fig. 5, decision curve analyses showed good clinical benefit, especially in the ExtraTrees model and the LightGBM model.

**Table 1:** WSI level performances for prognostic model of odontogenic keratocysts

Model	WSI	Cohort	AUC	95% CI	Accuracy	Sensitivity	Specificity
SVM	Multi	Training	0.943	0.905–0.982	0.914	0.864	0.924
		Testing	0.840	0.751–0.930	0.675	0.929	0.642
	Single	Training	0.979	0.952–1.000	0.979	0.955	0.983
		Testing	0.699	0.572–0.826	0.658	0.714	0.657
Random forest	Multi	Training	0.958	0.930–0.986	0.929	0.886	0.936
		Testing	0.811	0.707–0.914	0.683	0.857	0.667
	Single	Training	0.947	0.917–0.977	0.893	0.841	0.903
		Testing	0.692	0.567–0.817	0.650	0.786	0.638
ExtraTrees	Multi	Training	0.940	0.902–0.977	0.854	0.909	0.843
		Tesingt	0.825	0.727–0.924	0.708	0.857	0.689
	Single	Training	0.941	0.908–0.973	0.886	0.841	0.894
		Testing	0.656	0.531–0.782	0.475	0.929	0.419
XGBoost	Multi	Training	0.973	0.951–0.995	0.932	0.932	0.932
		Testing	0.787	0.667–0.907	0.642	0.929	0.610
	Single	Training	0.973	0.955–0.991	0.907	0.932	0.903
		Testing	0.629	0.477–0.780	0.617	0.714	0.610
LightGBM	Multi	Training	0.959	0.935–0.983	0.911	0.886	0.915
		Testing	0.819	0.725–0.913	0.692	0.857	0.683
	Single	Training	0.933	0.901–0.966	0.829	0.886	0.818
		Testing	0.598	0.442–0.755	0.600	0.714	0.596
MLP	Multi	Training	0.922	0.882–0.963	0.875	0.841	0.881
		Testing	0.798	0.689–0.907	0.617	0.929	0.575
	Single	Training	0.961	0.934–0.989	0.907	0.886	0.911
		Testing	0.613	0.485–0.741	0.483	0.857	0.434

*WSI* whole slide image, *SVM* support vector machines, *LightGBM* light gradient boosting machine, *MLP* multilayer perceptron

Simultaneously, we also aggregated information from a single slide of individual patients. When we aggregated these features and reapplied machine learning algorithms, there were significant differences in performance. Compared to the results at the patch-level, all single-slide WSI-level models showed better performance on the training dataset (Table 1), while only the SVM (AUC = 0.699, 95% CI: 0.572–0.826) and random forest (AUC = 0.692, 95% CI: 0.567–0.817) models showed improvement after aggregation at the single slide WSI-level on the testing dataset (Fig. 4c). As shown in Supplementary Fig. 6, the decision curve analyses demonstrated lower clinical benefit in all machine learning models of single slice WSI-level than at the multiple slides WSI-level.

To validate the difference between using multiple slides and a single slide, we compared these two SVM models. While the single-slide model demonstrated higher performance compared to multiple-slides model on the training dataset (Supplementary Fig. 4B), it significantly underperformed on the testing dataset (Fig. 4d). We also compared the two different AUC values using the DeLong test, resulting in *P* = 0.002 and *P* < 0.001 for the training and testing datasets, respectively. These *P* values are both less than 0.05, indicating a statistically significant difference in performance improvement. We suspect that the difference arises from the fact that, during feature aggregation, a single slice has less data to express its performance, potentially leading to overfitting on the training dataset and poorer performance on the testing dataset.

### Artificial intelligence features associated with pathological findings

**Fig. 5:**
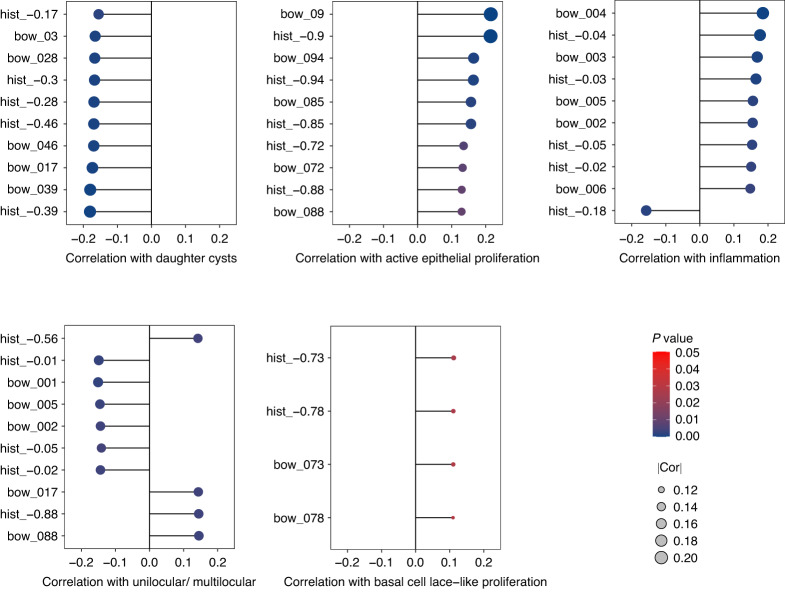
Artificial intelligence features associated with pathological findings. The correlation between pathology features generated by the AI model and the pathological findings observed on H&E-stained slides in 400 cases of OKC was analyzed. Among all AI features, “hist_-0.39”, “bow_039”, “bow_017”, “bow_046”, “hist_-0.46”, “hist_−0.28”, “hist_−0.3”, “bow_028”, “bow_03”, and “hist_−0.17” were the pathology features most correlated with daughter cysts. “Bow_09”, “hist_-0.9”, “bow_094”, “hist_−0.94”, “hist_-0.85”, “bow_085”, “hist_−0.72”, “bow_072”, “hist_−0.88”, and “bow_088” were the pathology features most correlated with active epithelial proliferation. “Bow_004”, “hist_-0.04”, “bow_003”, “hist_-0.03”, “hist_-0.18”, “bow_005”, “bow_002”, “hist_−0.05”, “hist_−0.02”, and “bow_006” were the pathology features most correlated with inflammation. “Bow_001”, “hist_−0.01”, “bow_005”, “bow_088”, “hist_-0.88”, “hist_−0.56”, “bow_002”, “hist_−0.02”, “hist_−0.05”, and “bow_017” were the pathology features most correlated with unilocular/multilocular. Only four features were correlated with basal cell lace-like proliferation: “hist_-0.73” (*P* = 0.025), “hist_-0.78” (*P* = 0.027), “bow_073” (*P* = 0.027), and “bow_078” (*P* = 0.028)

A total of 206 pathology features were generated by Inception_v3 in the prognostic model (Supplementary Table 2). We analyzed the correlation between pathology features generated by the artificial intelligence model and the pathological findings observed on H&E-stained slides in 400 cases of OKC. The pathological findings, such as daughter cysts, active epithelial proliferation, inflammation, unilocular/ multilocular, basal cell lace-like proliferation is shown in Supplementary Table 3. Among all AI features, 97 features were correlated with daughter cysts (*P* < 0.05). “Hist_-0.39”, “bow_039”, “bow_017”, “bow_046”, and “hist_-0.46” were the pathology features most correlated with daughter cysts. Eighteen features were correlated with active epithelial proliferation (*P* < 0.05). “Bow_09”, “hist_-0.9”, “bow_094”, “hist_-0.94”, and “hist_-0.85” were the pathology features most correlated with active epithelial proliferation. 53 features were correlated with inflammation (*P* < 0.05). “Bow_004”, “hist_-0.04”, “bow_003”, “hist_-0.03”, and “hist_-0.18” were the pathology features most correlated with inflammation. 90 features were correlated with unilocular/ multilocular (*P* < 0.05). “Bow_001”, “hist_-0.01”, “bow_005”, “bow_088”, and “hist_-0.88” were the pathology features most correlated with unilocular/multilocular. Only four features were correlated with basal cell lace-like proliferation: “hist_-0.73” (*P* = 0.025), “hist_-0.78” (*P* = 0.027), “bow_073” (*P* = 0.027), and “bow_078” (*P* = 0.028). The pathology features most correlated with these pathological findings are shown in Fig. 5.

## Discussion

OKC is a cystic lesion of the jaws that is considered benign. However, its high potential for proliferation results in a tendency to recur after surgery, indicating the necessity of its clinical management and regular follow-up.^2^ GS is recognized as a rare syndrome that is consisted of OKCs, multiple basal cell carcinomas, medulloblastoma, and a variety of skeletal and developmental abnormalities.^7^ GS exhibits a considerably greater inclination for recurrence after treatment when compared to sporadic OKC; hence, they cannot be regarded simply as the same entity.^5,6^ However, there was no significant difference between sporadic OKC and GS among mutations in the *PTCH1*, proliferating markers such as proliferating cell nuclear antigen (PCNA), Ki-67, and p53 expression, as well as broad-spectrum transcriptomic features.^33–36^ And so far, there is no clear method of identification when there was no clinical manifestations except OKC at the early stage of GS. OOC is a relatively uncommon odontogenic developmental cyst, and was previously classified as belonging to OKC. However, it needs to be differentiated from OKC because OOC lacks the *PTCH1* mutation and exhibits distinct histological characteristics and biological behavior, particularly its absence of recurrence following surgery.^9–11^

Computational pathology involves using deep learning methods and algorithms to analyze histopathological images.^37^ Due to the advancements in AI, there has been a surge in innovation in digital pathology. This ranges from automating routine diagnostic tasks to uncovering new prognostic and predictive biomarkers from histomorphology.^18–21,38,39^ Although pathologists are able to diagnose diseases through the examination of tissue samples and offer prognostic information by typing and grading the disease, the decision-making process heavily relies on complex visual features that require extensive training and skills.^37^ The prolonged training duration and escalated workload in histopathology raise concerns. In order to overcome these challenges, it is crucial to develop novel computational tools that can assist pathologists in carrying out routine diagnostic duties and thereby ease their onerous workload whilst potentially revealing fresh perspectives to bolster precision medicine.^37^ Moreover, the diagnostic efficiency of AI surpassed that of pathologists to a noteworthy extent. AI is capable of processing over 250 million images per day, which can be particularly beneficial in clinical management for heavily burdened diagnostic tasks and in areas with a shortage of pathologists.^40^ AI system utilizes deep learning methodologies to scrutinize WSIs, with the aim of forming algorithms that can undertake standard diagnostic procedures. This involves constructing models that meticulously examine tissue morphology in order to predict diagnoses or assess prognoses.^17,31,32,37^

**Fig. 6:**
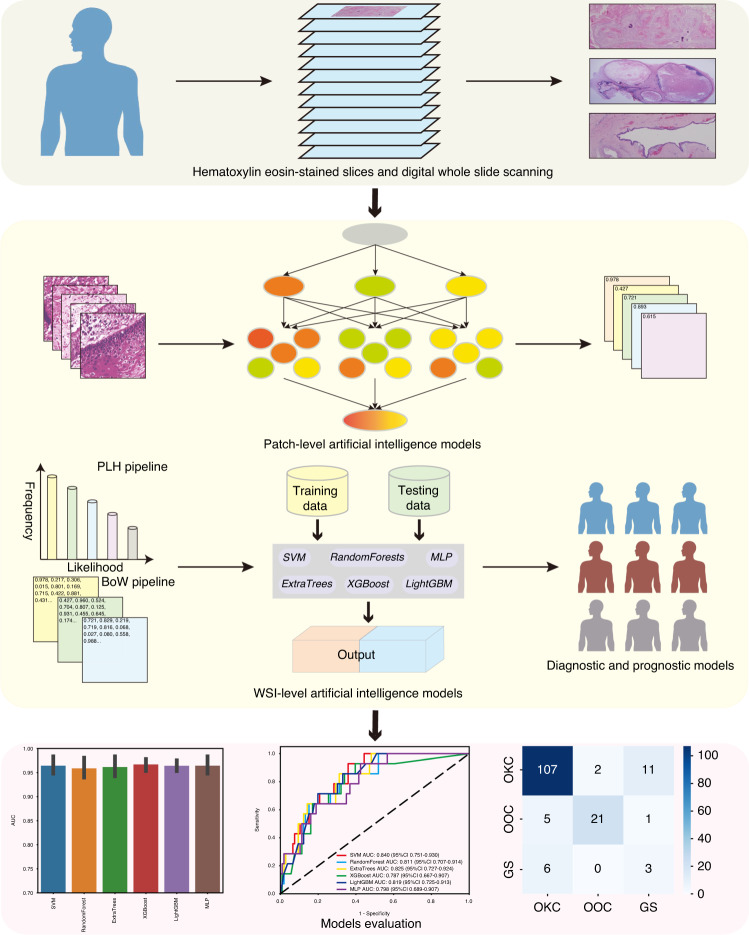
The workflow of artificial intelligence models development. Hematoxylin eosin-stained slices of various types of jaw cysts were collected and digital whole slide scanning. Next, Inception_v3 was employed to develop patch-level artificial intelligence model and multiple machine learning approaches were used to develop WSI-level artificial intelligence models. Finally, the performance of different AI models was evaluated in the testing dataset

The research has been carried out to differentiate between OKC and periapical cyst via machine learning recognition of computed tomography (CT) images and achieved 84.6%–96.0% accuracy with higher diagnostic accuracy for CT images compared to dental panoramic radiography.^41,42^ Interestingly, the Bouligand–Minkowski fractal descriptors using H&E staining histological slides even provided 100% accuracy rates.^43^ The differential diagnosis of OKC and ameloblastoma via deep learning recognition of CT images achieved identification accuracy of 84.6%–92.5%.^44,45^ Frydenlund et al. implemented an automated epithelial segmentation algorithm for H&E staining digital images to identify various cysts, including OKC, dentigerous cysts, lateral periodontal cysts, and glandular odontogenic cysts, subsequently realizing 90%–95% accuracy.^46^ The remarkable performance of AI model in identifying jaw cysts like OKC with periapical cysts and ameloblastoma could be attributed to the substantial disparities in their pathological and radiological features. However, when unable to detect typical GS attributes, such as basal cell carcinoma and palmar dyskeratosis, the differentiation of sporadic OKC and GS becomes complicated. A recent AI study only achieved an accuracy of only 68% to the differential diagnosis.^43^ It has been suggested that large lesions of OOC may be parallel to OKC radiologically, whereas common histopathological characteristics of OKC, including palisading basal cells and reverse nuclear polarity have not been identified.^10^ However, there is a lack of efficient and robust pathomics AI models for distinguishing between sporadic OKC, OOC, and GS rapidly. Therefore, it is imperative to develop digital pathology-based AI for identification of various types of jaw cysts. To the best of our knowledge, this is the first study to develop an objective diagnostic model to differentiate OKC from OCC and GS, using the largest sample size of OKC cohort currently available. The workflow of the AI model construction is shown in Fig. 6. The model presented high AUC of 0.935 (95% CI: 0.898–0.973), indicating accurate diagnosis of OKC from other diseases. Moreover, the results of the confusion matrix highlighted a greater differentiation between OKC and OCC in comparison with GS. The relationship between OKC and GS is strengthened by numerous correlations, such as similar *PTCH1* mutations and gene expression profiles, resulting in the confusion tendency.^34,36,47^

Due to the high recurrence rate of OKC, research has been directed towards identifying risk factors that may contribute to OKC recurrence. Although there have been reports of an increased proliferative potential in OKCs carrying *PTCH1* truncation-causing mutations, it has been found that the expression of COX-2, bcl-2, PCNA, and p53 were not linked to OKC recurrence.^12,15^ Additionally, the inflammatory pathological phenotype of OKC did not affect proliferative potential, and clinical, radiological, and histopathological parameters may only be potentially associated with OKC recurrence.^5,13,14,16^ Consequently, there is still a lack of a recognized model for the assessment of OKC recurrence. Here, we collected a substantial OKC cohort sample with an average follow-up duration of 3.6 years. The AI system-designed recurrence model for OKC demonstrates promising model performance (AUC = 0.840, 95% CI: 0.751–0.930) and application potential. We also analyzed the performance of the multi-slide fusion model and the single-slide model for recurrence assessment. The findings indicated that the multi-slide model performs significantly better than the single-slide model (AUC = 0.699, 95% CI: 0.572–0.826) on the testing dataset. As OKCs are typically surgically resected specimens with large cyst volumes, deep learning on a single slide may not capture representative information of the entire case, resulting in inadequate prognostic evaluations. For large-volume samples, such as cysts and tumors, a multi-slide fusion model needs to be performed to enable the AI system acquiring more learning information and incorporate it into the model construction. Furthermore, the correlation between AI-generated pathology features and pathological findings, such as daughter cysts, active epithelial proliferation, inflammation, unilocular/multilocular, basal cell lace-like proliferation was analyzed. Multiple AI features correlated significantly with pathological findings, indicating the interpretability of AI models based on digital pathology.

However, there are several limitations in this study. First, it solely investigated a single-center sample, and a multi-center cohort should be taken into account in the future to validate the model performance. Second, this study collected a retrospective cohort, and the prospective design would strengthen the findings of the study. Additionally, AI models are not static and must evolve in the future as algorithms are developed for AI-assisted healthcare systems. For example, the chat generative pre-trained transformer (ChatGPT) has potential for integration into healthcare systems assisted by artificial intelligence.

In summary, we developed the diagnostic and prognostic models for OKC through digital pathology-based artificial intelligence. Our AI models have demonstrated satisfactory performance in the testing cohort. Hence, the use of AI systems for clinical management of OKC can be considered in the future.

## Materials and methods

### Data collection

The flowchart for the cohorts used in this study is shown in Fig. 1. A total of 543 cases, encompassing OKC, OOC, and GS, were obtained from Peking University Hospital of Stomatology between 2000 and 2020. Of these, 24 cases were excluded due to unclear or faded H&E staining. The remaining 519 cases, along with a total of 2 157 H&E-stained slides, were then randomly assigned to the training and testing cohorts. The training cohort comprised 363 cases, while the testing cohort had 156 cases. This division was made in a 7:3 ratio to facilitate the development of the diagnostic model (Supplementary Table 4). Four hundred cases of OKC were randomly assigned into two groups: the training cohort (280 cases) and the testing cohort (120 cases), in order to develop a prognostic model of OKC using a ratio of 7:3. The baseline data of the prognostic model and diagnostic model were shown in Supplementary Tables 3 and 4. All H&E-stained slides were scanned using a NanoZoomer for digital whole slide imaging (WSI) and then exported to NDPI by NDPView2 software. The Institutional Ethics Board of Peking University Hospital of Stomatology approved this study.

### Data processing

To address the significant issue of managing extensive digitally processed images, we have implemented a systematic pre-processing strategy. This has involved segmenting WSIs into smaller 512 × 512 pixels tiles, resulting in over 2.5 million patches. The non-overlapping partitioning approach adhered strictly to a resolution of 0.5 μm/pixel. Our main objective during this process was to secure high-quality data. To achieve this objective, we utilized a white background removal tool of OnekeyAI platform that is based on deep learning models.

In addition, we applied the Macenko method to normalize the color of small tiles. Moreover, we employed Z-score normalization on the RGB channels to obtain a standard normal distribution of image intensities, which served as input for our model. During training process, we employed online data augmentations, such as randomly flipping horizontally and vertically. Nevertheless, during testing process, we solely utilized normalization.

### Deep learning training

Our deep learning process comprised two tiers of predictions: patch-level and WSI-level predictions. Considering the images’ significant size and diversity, we started by segmenting the WSIs into smaller patches. Subsequently, we utilized a multi-instance learning algorithm to consolidate the patch likelihoods, leading to the WSI-level prediction. As the diagnostic model and prognostic model have different purposes, we replicated comparable measures to model the information for these two discrete tasks.

For patch-level predictions, we evaluated the efficacy of the widely recognized neural network, Inception_v3. This convolutional neural network has displayed significant outcomes in the ImageNet classification contest. We aimed to establish the probability of each patch receiving the label corresponding to the respective WSI to which it pertained.

To improve the model’s ability to generalize across heterogeneous cohorts, we implemented transfer learning. This entailed initializing the model’s parameters with pretrained weights from the ImageNet dataset, while retaining the patch-level discriminators’ weights. Afterward, we fine-tuned the entire model using a limited dataset (training set with 363 samples) that had been specifically weakly annotated for our task. By utilizing transfer learning, we successfully utilized the knowledge obtained from ImageNet and tailored it to suit the requirements of our classification problem.

To improve generalization, we meticulously set the learning rate using the cosine decay learning rate algorithm in this study. The learning rate is presented as follows:

$${\eta }_{t}^{{task}-{spec}}={\eta }_{\min }^{i}+\frac{1}{2}\left({\eta }_{\max }^{i}-{\eta }_{\min }^{i}\right)\left(1+\cos \left(\frac{{T}_{{cur}}}{{T}_{i}}\pi \right)\right)$$

${\eta }_{\min }^{i}=0$, ${\eta }_{\max }^{i}=0.01$, ${T}_{i}=8$ represent the minimum learning rate, the maximum learning rate, and the number of iteration epochs, respectively. The use of a relatively small *T*_*i*_ is justified by our vast dataset, which comprises over 2.5 million training patches. We also utilize transfer learning algorithms to ensure optimal model fitting. As the backbone component already includes pre-trained parameters, fine-tuning is imperative for effective transfer. Therefore, we fine-tune the backbone component parameters when ${T}_{{cur}}=\frac{1}{2}{T}_{i}$. Furthermore, the learning rate for the backbone component is defined as follows:

$${\eta }_{t}^{{backbone}}=\left\{\begin{array}{ll}0 & \text{if}\,{T}_{{cur}}\le \frac{1}{2}{T}_{i}\\ {\eta }_{\min }^{i}+\frac{1}{2}\left({\eta }_{\max }^{i}-{\eta }_{\min }^{i}\right)\left(1+\cos \left(\frac{{T}_{{cur}}}{{T}_{i}}\pi \right)\right) & \text{if}\,{T}_{{cur}} > \frac{1}{2}{T}_{i}\end{array}\right.$$

Other hyperparameter configurations are as follows: optimizer—SGD, loss function—softmax cross-entropy, with a batch size of 128. We use the Gridsearch algorithm to search for classical model parameters such as n_estimator and max_depth. In practice, our n_estimator is searched from 10 to 50 with a compensation of 5. max_depth is searched for 2, 3, 4, and 5 to form 40 corresponding search models.

### Multi-instance learning for WSI fusion

After the training of our deep learning model, we carried out label predictions and their respective probabilities for all patches. A classifier was then used to aggregate these patch probabilities, resulting in a WSI-level prediction. To collect the patch likelihoods, we made use of two different machine-learning methods:

Patch Likelihood Histogram (PLH) pipeline: In this approach, we used a histogram to represent the distribution of patch likelihoods within the WSI. By discretizing the likelihoods and retaining only one decimal place in the development of diagnostic model, and two decimal places in the development of prognostic model, we effectively captured the distribution of likelihoods, which served as a representation of the WSI.

Bag of Words (BoW) pipeline: Building on both histogram-based and vocabulary-based techniques, the BoW pipeline utilized a term frequency-inverse document frequency (TF-IDF) mapping for every patch, which resulted in TF-IDF feature vectors that represented the WSIs. These feature vectors were subsequently employed for training conventional machine learning classifiers to predict the status in each WSI.

By deploying two independent pipelines, we successfully amalgamated the initially dispersed patch-level predictions, producing WSI-level features. These features furnish significant information for subsequent analytical operations.

### Signature building

In this study, final patient representations were constructed utilizing patch-level predictions, probability histograms, and TF-IDF features in combination. Initially, a *t* test statistical analysis was carried out to pinpoint statistically significant pathology features with the purpose of refining the feature selection process for both diagnostic model and prognostic model. Then we utilized machine learning algorithms, such as support vector machines (SVM), tree-based models, such as random forests and extremely randomized trees (ExtraTrees), extreme Gradient Boosting (XGBoost), and light gradient boosting machine (LightGBM), as well as multilayer perceptron (MLP), to develop our models. Each model is explained in further detail below:

Random forest is an integrated learning technique that generates predictions by constructing and merging numerous decision trees. The number of trees in the forest is defined by the parameter of n_estimatores, while max_depth determines the maximum depth of the tree. Additionally, the minimum number of samples required to split the internal nodes is defined by the min_samples_split.

XGBoost is an optimized distributed gradient boosting library that implements state-of-the-art gradient boosting algorithms. The model’s learning and optimization procedures can be regulated by means of parameters such as n_estimatores, max_depth and min_child_weight.

LightGBM is another gradient-boosting framework that employs decision trees as a base learner. The maximum depth of each tree is controlled by max_depth and n_estimatores to regulate the number of learners.

ExtraTrees is a variation of random forest with an increased degree of freedom to explore the parameter space more effectively during the training process. The parameters are similar to those used in random forest.

SVM uses the RBF kernel function, while the other parameters are kept as default. MLP is a fully connected 3-layer perceptron, comprises 128, 64, and 32 hidden nodes, respectively. All of these models employ an implementation of scikit-learn, a widely used machine learning library in Python data science.

### Model evaluation

To validate the accuracy of the pathology model in region identification, we carried out a comprehensive assessment using receiver operating characteristic (ROC) curves at patch level. The aggregation of patches into WSI was visualized for performance evaluation, which included predicted labels and probability heatmaps for the patches. For the diagnostic model, we utilized both micro and macro area under the curve (AUC) metrics to achieve a holistic evaluation of the model performance. Additionally, we employed the “One vs. Others” strategy to evaluate the AUC for each prediction class. Confusion matrices were also utilized to assess the model performance. For the prognostic model, we used AUC as the performance metric and calculating sensitivity and specificity. Furthermore, we compared the performance of single-slice and multi-slices fusion models using Delong’s test to measure significance. The study employed a range of software tools, including ITK SNAP v.3.8.0, and custom Python code written in Python v.3.7.12. Python packages used in the analysis included Pandas v.1.2.4, NumPy v.1.20.2, PyTorch v.1.8.0, Onekey v.2.2.3, OpenSlide v.1.2.0, Seaborn v.0.11.1, Matplotlib v.3.4.2, SciPy v.1.7.3, scikit-learn v.1.0.2, PyRadiomics v.3.0.

### Supplementary information

Supplementary Figure 1

Supplementary Figure 2

Supplementary Figure 3

Supplementary Figure 4

Supplementary Figure 5

Supplementary Figure 6

Supplementary Tables 1, 3, and 4

Supplementary Table 2
