# Supplementary material for: Digital pathology-based artificial intelligence models for differential diagnosis and prognosis of sporadic odontogenic keratocysts
Source: Int J Oral Sci. 2024 Feb 26;16:16. doi: 10.1038/s41368-024-00287-y (PMC10894880; doi:10.1038/s41368-024-00287-y)
Supplement: Supplementary file 1 — Supplementary Figure 1 [file 41368_2024_287_MOESM1_ESM.pdf]

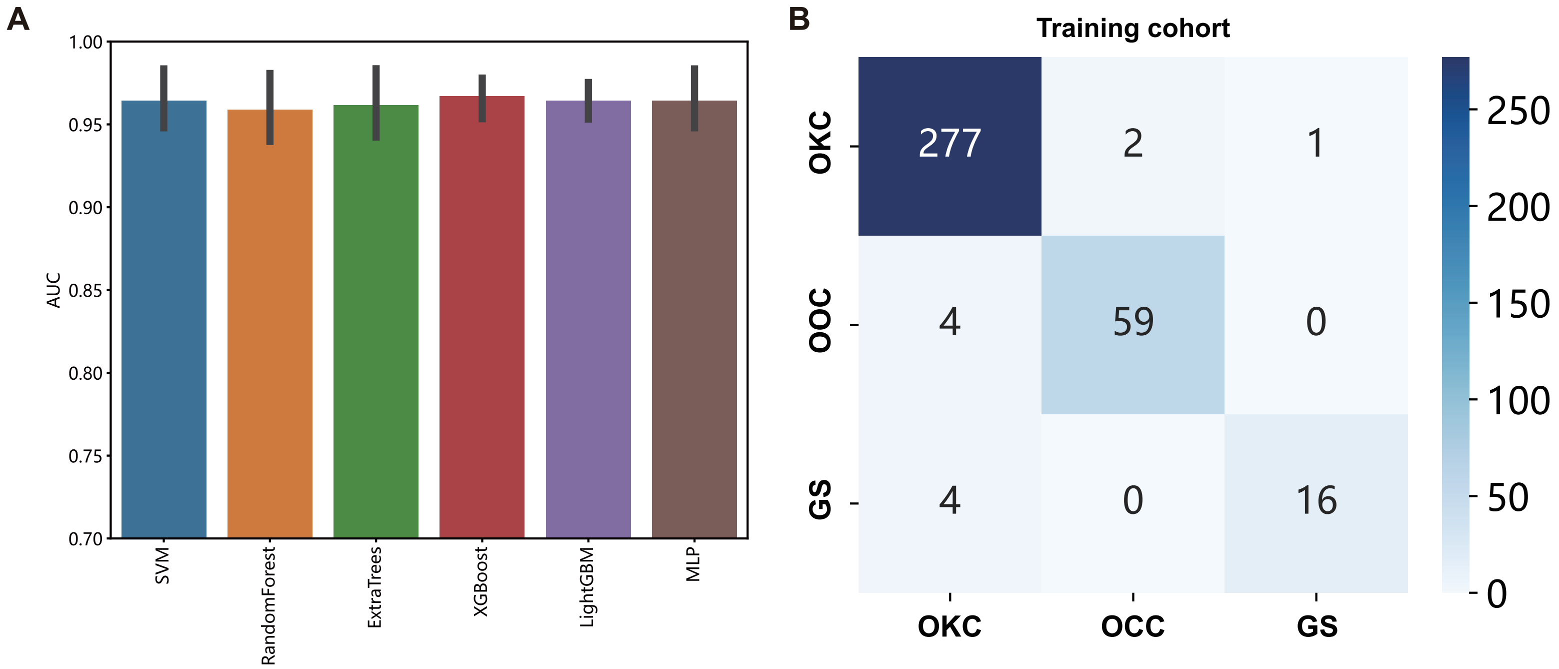

Supplementary Figure 1. (A) The cross-validation on the training dataset indicated good performances for all models. (B) The confusion matrices for the training datasets to visually interpret the model classification performance.
