# Supplementary material for: Digital pathology-based artificial intelligence models for differential diagnosis and prognosis of sporadic odontogenic keratocysts
Source: Int J Oral Sci. 2024 Feb 26;16:16. doi: 10.1038/s41368-024-00287-y (PMC10894880; doi:10.1038/s41368-024-00287-y)
Supplement: Supplementary file 4 — Supplementary Figure 4 [file 41368_2024_287_MOESM4_ESM.pdf]

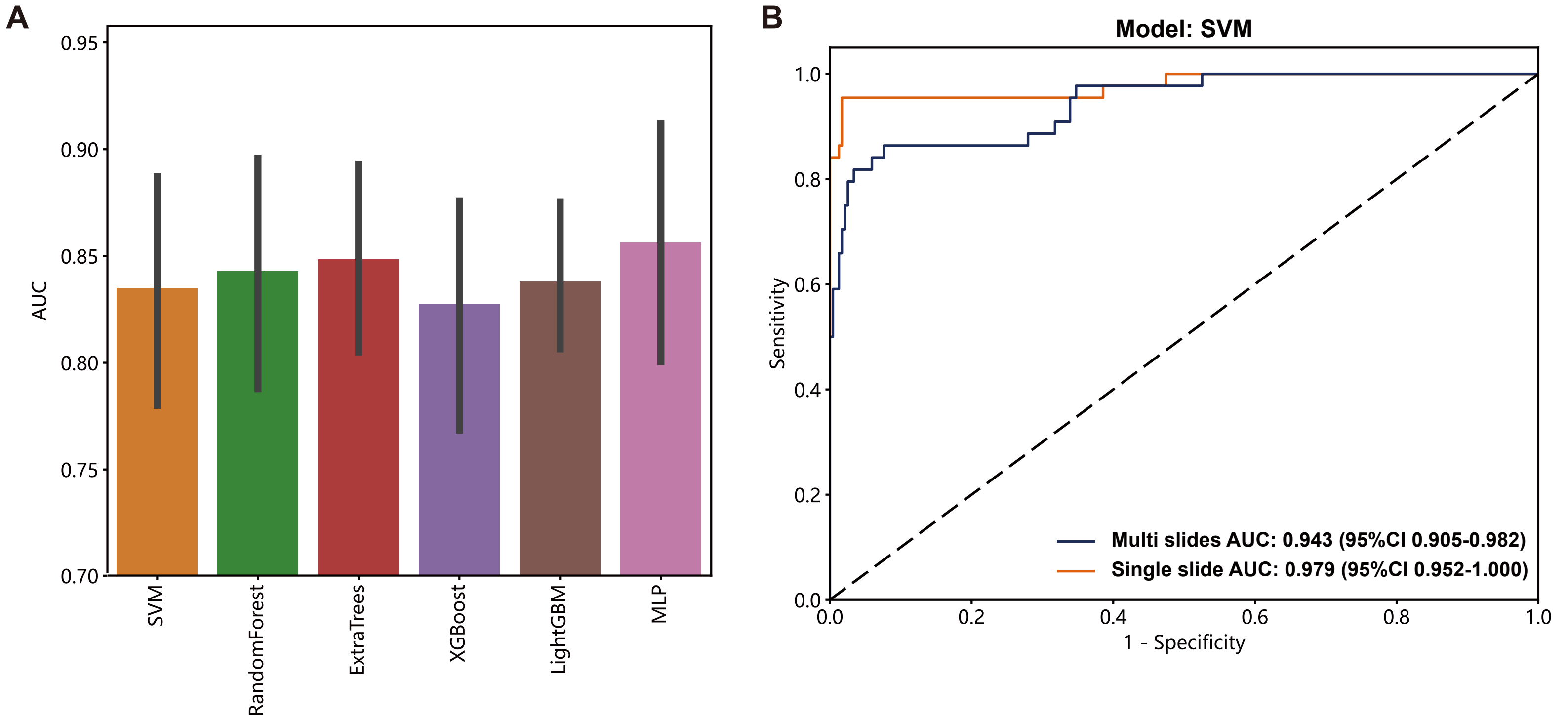

Supplementary Figure 4. (A) The results of 5-fold cross-validation on the training dataset. (B) The comparison between multiple-slide and single-slide SVM model on the training dataset.
