# Supplementary material for: Digital pathology-based artificial intelligence models for differential diagnosis and prognosis of sporadic odontogenic keratocysts
Source: Int J Oral Sci. 2024 Feb 26;16:16. doi: 10.1038/s41368-024-00287-y (PMC10894880; doi:10.1038/s41368-024-00287-y)
Supplement: Supplementary file 6 — Supplementary Figure 6 [file 41368_2024_287_MOESM6_ESM.pdf]

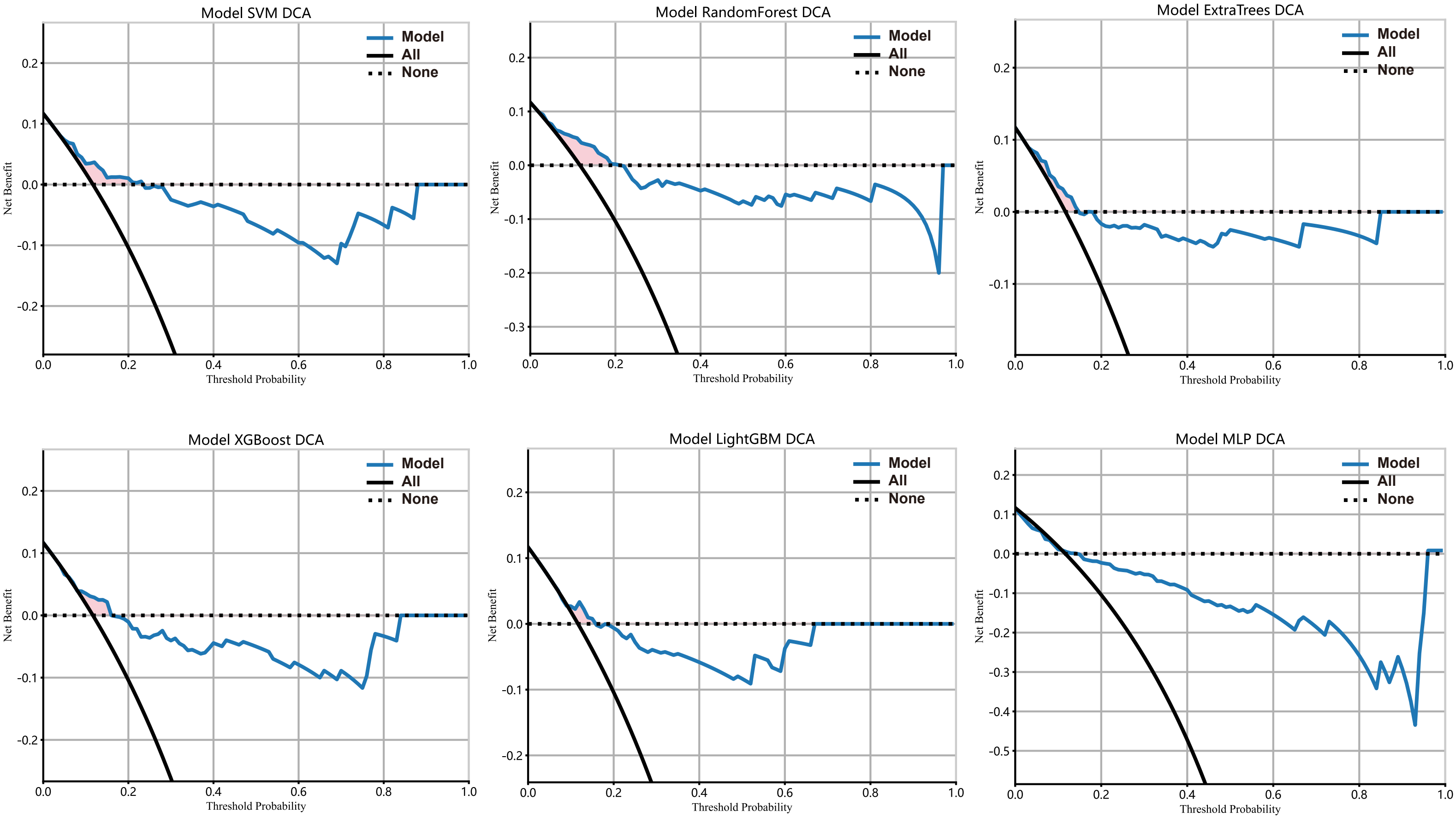

Supplementary Figure 6. Decision curve analyses among different machine learning methods in the testing cohort of single-slide prognostic models.
