# Supplementary material for: Digital pathology-based artificial intelligence models for differential diagnosis and prognosis of sporadic odontogenic keratocysts
Source: Int J Oral Sci. 2024 Feb 26;16:16. doi: 10.1038/s41368-024-00287-y (PMC10894880; doi:10.1038/s41368-024-00287-y)
Supplement: Supplementary file 7 — Supplementary Tables 1, 3, and 4 [file 41368_2024_287_MOESM7_ESM.docx]

Supplementary Table 1. Patch level (Inception_v3) and WSI level (SVM) performances of diagnostic model

| Model | Cohort | AUC | 95% CI | Accuracy | Sensitivity | Specificity |
| --- | --- | --- | --- | --- | --- | --- |
| Inception_v3 | training-OKC | 0.868 | 0.867-0.868 | 0.787 | 0.796 | 0.755 |
|  | training-OOC | 0.883 | 0.882-0.884 | 0.822 | 0.743 | 0.838 |
|  | training-GS | 0.793 | 0.792-0.794 | 0.652 | 0.772 | 0.643 |
|  | testing-OKC | 0.757 | 0.755-0.758 | 0.786 | 0.885 | 0.559 |
|  | testing-OOC | 0.866 | 0.865-0.867 | 0.854 | 0.694 | 0.901 |
|  | testing-GS | 0.474 | 0.472-0.477 | 0.911 | 0.027 | 0.985 |
| SVM | training-OKC | 0.975 | 0.948-1.000 | 0.978 | 0.982 | 0.964 |
|  | training-OOC | 0.999 | 0.998-1.000 | 0.994 | 1.000 | 0.993 |
|  | training-GS | 0.988 | 0.975-1.000 | 0.909 | 1.000 | 0.904 |
|  | testing-OKC | 0.935 | 0.898-0.973 | 0.853 | 0.825 | 0.944 |
|  | testing-OOC | 0.989 | 0.976-1.000 | 0.917 | 1.000 | 0.899 |
|  | testing-GS | 0.811 | 0.664-0.959 | 0.712 | 0.889 | 0.705 |

SVM: support vector machines.

Supplementary Table 3. Baseline data of prognostic model of odontogenic keratocysts

| Data sets | Training cohort | Testing cohort |
| --- | --- | --- |
| No. | 280 | 120 |
| Age | 35.28 ± 16.22 | 36.31 ± 16.37 |
| Sex |  |  |
| Male | 144 (51.43%) | 66 (55.00%) |
| Female | 136 (48.57%) | 54 (45.00%) |
| Site |  |  |
| Maxilla | 74 (26.43%) | 43 (35.83%) |
| Mandible | 206 (73.57%) | 77 (64.17%) |
| Recurrence | 44 (15.71%) | 14 (11.67%) |
| Daughter cysts |  |  |
| No | 222 (79.29%) | 95 (79.17%) |
| Yes | 58 (20.71%) | 25 (20.83%) |
| Active epithelial proliferation |  |  |
| No | 273 (97.50%) | 117 (97.50%) |
| Yes | 7 (2.50%) | 3 (2.50%) |
| Inflammation |  |  |
| No | 26 (9.29%) | 6 (5.00%) |
| Yes | 254 (90.71%) | 114 (95.00%) |
| Unilocular/ multilocular |  |  |
| Unilocular | 193 (68.93%) | 89 (74.17%) |
| Multilocular | 87 (31.07%) | 31 (25.83%) |
| Basal cell lace-like proliferation |  |  |
| No | 274 (97.86%) | 110 (91.67%) |
| Yes | 6 (2.14%) | 10 (8.33%) |

Supplementary Table 4. Baseline data of diagnostic model

| Data sets | Training cohort | Testing cohort |
| --- | --- | --- |
| No. | 363 | 156 |
| Age | 34.95 ± 16.06 | 36.14 ± 16.69 |
| Sex |  |  |
| Male | 200 (55.10%) | 86 (55.13%) |
| Female | 163 (44.90%) | 70 (44.87%) |
| Site |  |  |
| Maxilla | 88 (24.24%) | 50 (32.05%) |
| Mandible | 275 (75.76%) | 106 (67.95%) |
| Diagnosis |  |  |
| Odontogenic keratocysts | 280 (77.13%) | 120 (76.92%) |
| Orthokeratinized odontogenic cyst | 63 (17.36%) | 27 (17.31%) |
| Gorlin syndrome | 20 (5.51%) | 9 (5.77%) |
